# Research Progress in the Detection of Mycotoxins in Cereals and Their Products by Vibrational Spectroscopy

**DOI:** 10.3390/foods14152688

**Published:** 2025-07-30

**Authors:** Jihong Deng, Mingxing Zhao, Hui Jiang

**Affiliations:** School of Electrical and Information Engineering, Jiangsu University, Zhenjiang 212013, China; 2112307126@stmail.ujs.edu.cn (J.D.); 15895187883@163.com (M.Z.)

**Keywords:** non-destructive testing, vibrational spectroscopy analysis technology, grains, mycotoxins

## Abstract

Grains and their derivatives play a crucial role as staple foods for the global population. Identifying grains in the food chain that are free from mycotoxin contamination is essential. Researchers have explored various traditional detection methods to address this concern. However, as grain consumption becomes increasingly time-sensitive and dynamic, traditional approaches face growing limitations. In recent years, emerging techniques—particularly molecular-based vibrational spectroscopy methods such as visible–near-infrared (Vis–NIR), near-infrared (NIR), Raman, mid-infrared (MIR) spectroscopy, and hyperspectral imaging (HSI)—have been applied to assess fungal contamination in grains and their products. This review summarizes research advances and applications of vibrational spectroscopy in detecting mycotoxins in grains from 2019 to 2025. The fundamentals of their work, information acquisition characteristics and their applicability in food matrices were outlined. The findings indicate that vibrational spectroscopy techniques can serve as valuable tools for identifying fungal contamination risks during the production, transportation, and storage of grains and related products, with each technique suited to specific applications. Given the close link between grain-based foods and humans, future efforts should further enhance the practicality of vibrational spectroscopy by simultaneously optimizing spectral analysis strategies across multiple aspects, including chemometrics, model transfer, and data-driven artificial intelligence.

## 1. Introduction

Grains represent the most essential staple food, with their safety directly impacting consumer health and social stability [1,2]. From field cultivation to final consumer products, cereals are exposed to potential safety risks throughout their supply chain. Practically, the safety of grains is currently limited by four main types of contamination: microbiological, chemical, physical and radioactive [3,4]. Microbial contamination includes common pathogens, viruses, and fungi [5,6]. Chemical contamination involves pesticide residues, heavy metals, harmful processing by-products, and illegally added substances [7,8,9]. Physical contamination mainly refers to foreign materials introduced during production and processing, such as metal fragments, plastic particles, and glass shards [10]. In addition, radioactive contamination caused by radioisotopes such as caesium-137 (Cs-137) and strontium-90 (Sr-90) is increasingly becoming a major issue in terms of grain safety. Among these, fungal contamination is particularly concerning due to its hidden onset, detection challenges, and long-lasting hazards, warranting special attention.

Mycotoxins are secondary metabolites produced by certain fungi under suitable temperature and humidity conditions. They exhibit high chemical stability and heat resistance, making them difficult to remove once present in food, even after thermal processing or physical treatments [11,12,13]. Major mycotoxins of regulatory concern include aflatoxins (AFs), ochratoxins (OTs), fumonisins (FUMs), deoxynivalenol (DON), and zearalenone (ZEN), which are commonly found in grains, nuts, dried fruits, spices, dairy products, and animal feed [14,15,16]. These toxins can form at various stages from cultivation to storage and transport. Their toxicological effects include chronic toxicity, carcinogenicity, immunosuppression, and teratogenicity, posing serious health risks with long-term low-dose exposure [17,18]. Aflatoxin B1 (AFB1) is classified by the International Agency for Research on Cancer (IARC) as a Group 1 carcinogen linked to liver cancer [19]. At the same time, ochratoxin A (OTA) is associated with kidney toxicity and potential carcinogenicity [20]. DON and ZEN disrupt protein synthesis and hormonal balance, adversely affecting the immune and reproductive systems [21]. Therefore, efficient and accurate detection of mycotoxins in food is essential to strengthen food safety management and protect public health.

The severity and prevalence of mycotoxin contamination have long drawn scientific attention. Over recent decades, numerous detection methods have been developed, with chromatographic analyses, immunoassays, and various rapid screening tools among the most widely used. Early detection mainly relied on thin-layer chromatography (TLC), whose simplicity met basic needs at the time [22,23]. However, as regulatory limits tightened and demands for higher sensitivity and accuracy increased, the limitations of TLC became apparent. High-performance liquid chromatography (HPLC), often coupled with immunoaffinity column (IAC) purification, was subsequently introduced, significantly improving detection and becoming a routine laboratory method [24,25]. Meanwhile, enzyme-linked immunosorbent assays (ELISA) have been adopted for large-scale screening during food processing and distribution [26,27]. In recent years, HPLC and liquid chromatography–tandem mass spectrometry (LC–MS/MS) have become the mainstream confirmatory techniques, recognized as the “gold standard” for quantitative mycotoxin analysis. They enable simultaneous detection of multiple toxins with high sensitivity, accuracy, and specificity. Nonetheless, these conventional methods often involve complex procedures, long processing times, and destructive sampling, limiting their application for rapid, non-destructive, online detection in food production [28,29,30]. Consequently, portable rapid tests have emerged, supporting on-site monitoring to some extent. For example, portable immunochromatographic strips (such as colloidal gold assays) can detect toxins at certain levels, meeting some real-time needs. However, their sensitivity and accuracy still fall short of stringent food safety standards.

Mycotoxin detection technologies face new demands for rapid screening, large-scale monitoring, and online real-time analysis. Although traditional methods perform well in sensitivity and accuracy, their complexity, long processing times, and destructive sampling expose clear limitations in modern food production and distribution [31,32,33]. In recent years, vibrational spectroscopy has attracted growing interest due to its minimal sample preparation, non-destructive nature, fast response, and potential for online integration, making it an important emerging approach for mycotoxin detection. Mycotoxin contamination is often accompanied by microscopic physicochemical changes in food matrices, such as protein, lipid, and carbohydrate composition shifts. By capturing spectral signals from molecular vibrations, vibrational spectroscopy can detect these changes and indirectly identify toxin contamination [34,35,36]. This technique is also highly adaptable and applicable to a wide range of food matrices, including solids, liquids, and powders. Furthermore, advances in chemometrics and machine learning have greatly improved the interpretation of spectral data, enhancing modeling accuracy and generalization, and providing strong technical support for rapid, non-destructive detection of mycotoxins in complex food systems. As a result, research on applying vibrational spectroscopy to mycotoxin detection continues to grow. Studies have successfully employed this technique to detect aflatoxins, ochratoxins, deoxynivalenol, and other toxins in corn, wheat, nuts, and similar matrices, achieving promising outcomes.

Accordingly, this paper reviews recent studies on the application of vibrational spectroscopy in mycotoxin detection, systematically summarizes various vibrational spectroscopy techniques for detecting typical toxins in different food matrices, identifies key challenges in current research, and discusses future directions for technological development. The structure of this paper is as follows: Section 2 outlines the basic principles and applicability of vibrational spectroscopy; Section 3 introduces standard chemometric and multivariate analysis methods used in spectral data processing; Section 4 provides a systematic summary of the application progress of different vibrational spectroscopy techniques in mycotoxin detection; Section 5 analyzes differences in applying these techniques to fungal evaluation in grains; Section 6 examines existing technical bottlenecks and explores future trends; finally, Section 7 concludes the paper.

## 2. Basic Principles of Vibrational Spectroscopy

**Figure 1:**
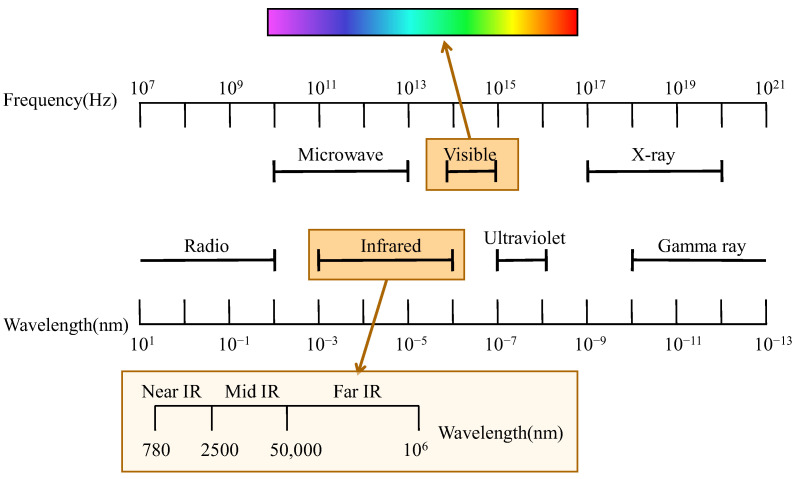
Wavelength range of different vibrational spectra.

Vibrational spectroscopy acquires chemical and physical information about a sample by detecting the absorption or scattering of electromagnetic waves during molecular vibrations or rotations [37,38,39]. Based on the spectral regions involved (as illustrated in Figure 1), vibrational spectroscopy can be categorized into visible–near-infrared (Vis–NIR), near-infrared (NIR), mid-infrared (MIR), Raman spectroscopy, and hyperspectral imaging (HSI). The types of molecular vibrational information provided by these spectral techniques vary, which determines their specific features and suitability in food analysis.

### 2.1. Vis–NIR

Vis–NIR covers the wavelength range of 400–1000 nm, spanning the visible region (400–780 nm) and the shortwave NIR region (780–1000 nm). Within this range, spectral signals primarily arise from electronic transition absorptions in the visible region and overtone or combination band absorptions of functional groups such as O–H, C–H, and N–H in the NIR region [40,41,42]. A distinct advantage of Vis–NIR is its ability to directly reflect color changes in food appearance, owing to electronic transitions of pigment compounds such as carotenoids, chlorophyll, and flavonoids. Thus, Vis–NIR is sensitive to macroscopic color attributes and microscopic molecular vibrations [43,44,45]. Generally, mycotoxin contamination is accompanied by increased fungal metabolic activity, leading to physicochemical changes in the food matrix, including the accumulation of pigment metabolites. These processes alter color in the visible region and cause shifts in NIR spectral features [46,47,48]. For these reasons, Vis–NIR spectroscopy has been applied to rapidly screen for mycotoxin contamination in grains and nut-based products.

### 2.2. NIR

**Figure 2:**
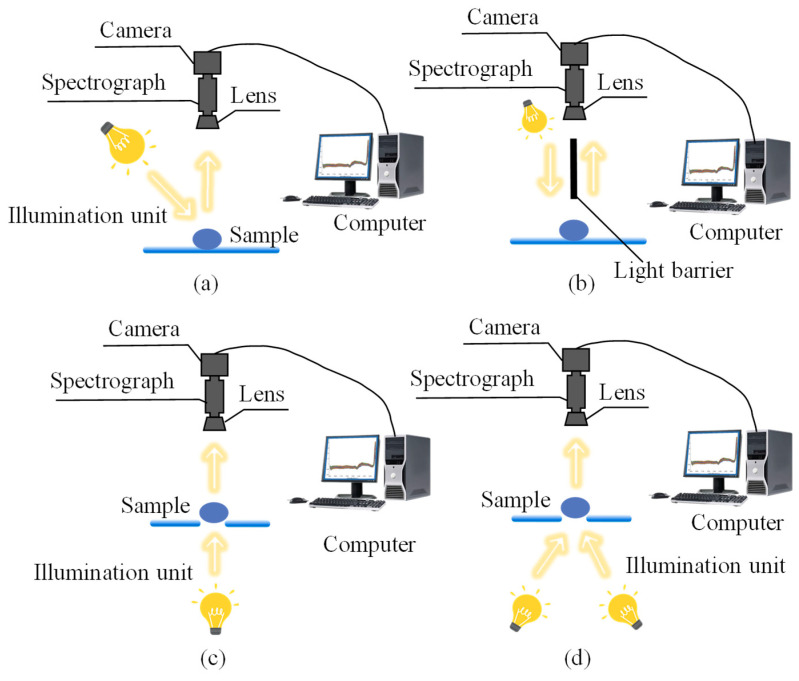
Data acquisition methods for NIR spectroscopy and hyperspectral systems. (**a**) Diffuse reflectance. (**b**) Interactance. (**c**) Full transmittance. (**d**) Partial transmittance.

NIR, spanning 780–2500 nm between the visible and mid-infrared regions, detects characteristic absorptions from hydrogen-containing groups, primarily due to overtone and combination vibrations of C–H, O–H, and N–H bonds [49,50,51,52,53,54]. When NIR light interacts with a sample, it undergoes transmission or reflection, enabling selective absorption by specific bonds, as shown in Figure 2. The resulting spectra provide qualitative and quantitative insights into the sample’s chemical composition and structure. Compared to Vis–NIR, NIR is less influenced by surface color and better suited for evaluating the internal quality of granular, powdered, and solid foods. To address the slow scanning, low signal-to-noise ratio, and mechanical noise of traditional dispersive instruments, Fourier-transform NIR (FT-NIR) employs a Michelson interferometer to capture full-range interferograms, later processed by Fourier transformation into high-resolution spectra [55,56,57]. This enhances signal quality and wavelength precision while minimizing light source and mechanical variability, making it highly effective for detecting subtle compositional changes in complex food matrices. Today, NIR and FT-NIR are applied to detect mycotoxins in grains rapidly.

### 2.3. MIR

MIR covers the 2500–25,000 nm range. This region corresponds to fundamental molecular vibrations and rotations [58,59,60]. Unlike NIR, which measures overtones and combination bands, MIR directly detects fundamental absorptions of functional groups. It produces distinct, particular peaks, often called the “molecular fingerprint region” [61]. In food samples, functional groups such as C=O, C–O, C=C, O–H, N–H, and P=O show clear absorptions in this range. This provides detailed information on chemical composition and molecular structure [62,63]. Compared to NIR, MIR offers stronger structural specificity. It supports more interpretable models with clear physicochemical meaning. However, MIR has a limited penetration depth, typically only a few to tens of micrometers. It is also sensitive to surface conditions [64,65]. Therefore, MIR often uses transmission to analyze liquids or thin films. For solids or viscous samples, attenuated total reflectance (ATR) is preferred. ATR requires minimal sample preparation and is simple to operate. It is now widely applied in analyzing complex food matrices.

### 2.4. Raman

**Figure 3:**
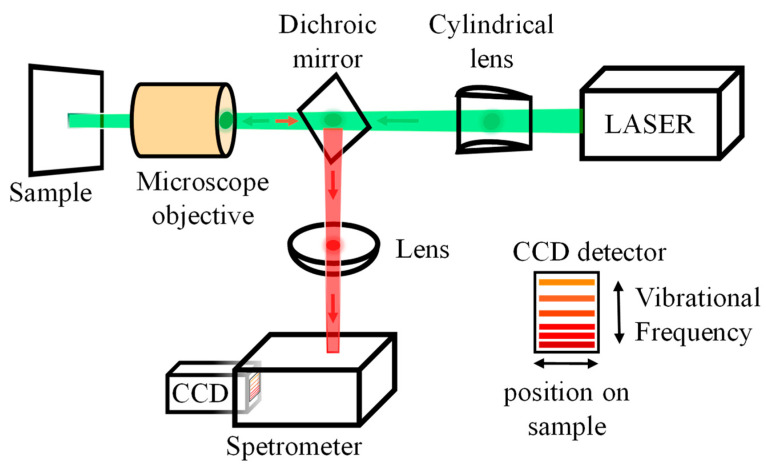
Classical Raman spectrum acquisition system.

Raman spectroscopy is also a valuable technique for analyzing food components. It is based on inelastic scattering (Raman scattering) when light interacts with matter [66,67]. Typically, Raman spectra cover 4000–50 cm^−1^, with 1800–400 cm^−1^ known as the fingerprint region, helpful in analyzing organic molecules and complex matrices [68]. Figure 3 illustrates the components of a Raman acquisition system. Unlike infrared methods, Raman spectroscopy is not sensitive to water content. It is particularly suited for high-moisture or liquid food systems, requiring no drying and enabling rapid, non-destructive sampling. Thus, Raman and NIR are highly complementary. However, because Raman scattering is a low-probability event, conventional Raman struggles with weak signals when detecting low-concentration targets [69]. To address this, surface-enhanced Raman spectroscopy (SERS) was developed. SERS enhances signals by adsorbing samples onto metal surfaces with nanoscale roughness, such as silver or gold nanoparticles. This can increase sensitivity by 10^4^–10^6^ times or more, enabling trace detection [70].

### 2.5. HSI

**Figure 4:**
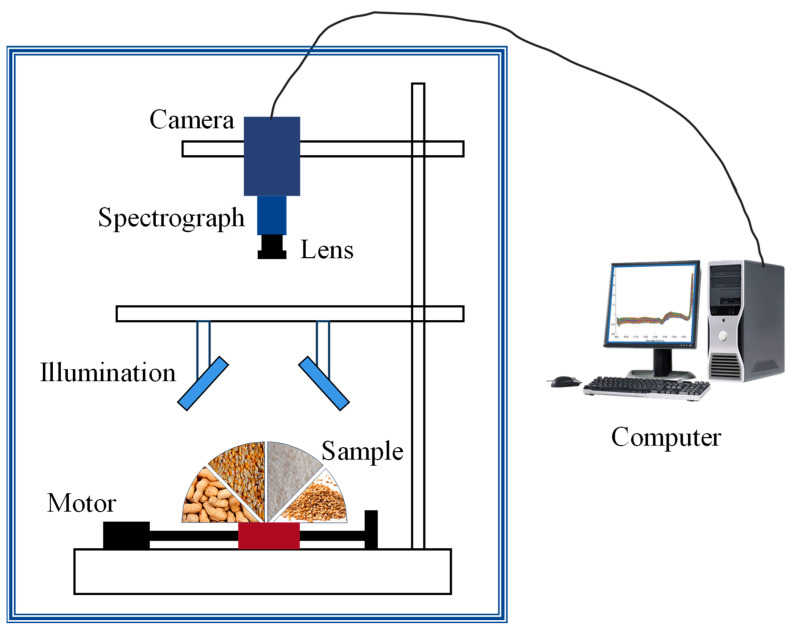
Classical HSI acquisition system.

Unlike the previously mentioned spectral techniques, hyperspectral imaging technology is a non-destructive analytical technique that can simultaneously provide both spectral information and spatial images, based on traditional computer vision [71,72,73]. Figure 4 shows a typical hyperspectral imaging system that includes different measurement modes, usually equipped with an illumination unit, spectrometer, high-performance camera, and a translation stage with a stepper motor and computer. By scanning the sample using this system, a hyperspectral image of the sample can be obtained. Each pixel in the hyperspectral image contains a spectrum, so the data obtained from this system is a three-dimensional data cube, which includes two-dimensional spatial information and the third dimension of spectral information. In practical applications, hyperspectral systems can generally be classified into different types based on their application, namely ultraviolet hyperspectral, visible–near-infrared hyperspectral, near-infrared or short-wave near-infrared hyperspectral, fluorescence hyperspectral, and Raman hyperspectral.

## 3. Spectral Analysis Framework

**Figure 5:**
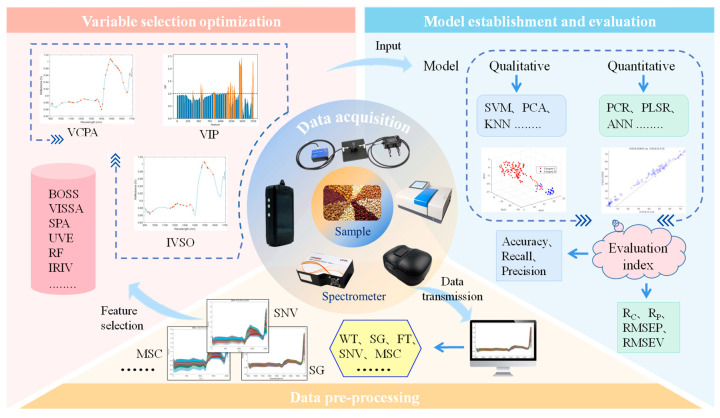
Spectral analysis framework.

The widespread use of vibrational spectroscopy in food composition analysis is largely attributed to advances in chemometrics. As an indirect measurement technique, vibrational spectroscopy often cannot directly link spectral features to target properties. Chemometric methods are essential to calibrate and model raw spectral data, extracting information related to the attributes of interest. In essence, the core aim of chemometrics is to uncover the intrinsic relationship between spectral data and sample properties, enabling robust predictive models. With the growing application of vibrational spectroscopy in food analysis, data processing workflows have become increasingly standardized and are widely accepted by researchers (see Figure 5). Common workflows typically include sample preparation, spectral data acquisition, preprocessing, feature extraction and variable selection, model building and evaluation, and result interpretation and practical application.

### 3.1. Sample Preparation and Data Acquisition

Sample preparation and data acquisition are essential for obtaining reliable spectral signals. Different vibrational spectroscopy techniques have distinct requirements regarding sample form and measurement conditions. Due to strong water absorption, MIR spectroscopy is generally applied to dry samples or thin films using ATR mode, and often avoids glass containers to reduce interference [74,75]. In contrast, Raman spectroscopy is less affected by water, allowing direct analysis of liquids and collection through capillaries or glass. NIR and Vis–NIR spectroscopy are widely used for non-destructive analysis of solids or powders. These methods benefit from uniform, flat sample surfaces, often requiring grinding or sieving to minimize scattering. HSI is particularly sensitive to surface texture, illumination, and spatial uniformity, necessitating strict control of imaging geometry and lighting conditions.

### 3.2. Dataset Partitioning

Dataset partitioning is the first formal step in spectral modeling. The calibration set should span the full range of the target attribute to ensure model generalization, while the test set must include unseen but representative samples to evaluate prediction performance. Common splitting strategies include random splitting, suitable for large, well-distributed datasets; the Kennard-Stone (KS) algorithm, which selects spectrally diverse samples based on Euclidean distance; and sample set partitioning based on joint x-y distance (SPXY), which considers both spectral and target variable distributions, particularly effective for heterogeneous datasets [76,77]. In practice, multiple methods are often compared to ensure model robustness.

### 3.3. Preprocessing Methods

Preprocessing is a key step in spectral analysis, aiming to minimize noise and systematic errors to enhance data quality and modeling accuracy. Raw spectra often suffer from interference caused by light scattering due to particle size or density differences, baseline drift resulting from optical instability, and background noise originating from complex food matrices [78,79]. Various preprocessing techniques are commonly applied to address these issues based on their specific functions. Smoothing methods such as the Savitzky–Golay (SG) filter are widely used to reduce high-frequency noise [80]. Baseline correction helps eliminate signal drift and improve spectral peak resolution [81]. Scatter correction techniques, including standard normal variate (SNV) and multiplicative scatter correction (MSC), are designed to compensate for physical variability between samples [82]. Additionally, mean centering and normalization standardize intensity scales, helping to reduce the effects of concentration differences across samples. In complex food systems, a single method is often insufficient. As a result, combined preprocessing strategies have become standard practice, typically tailored to the sample type and spectroscopic technique used.

### 3.4. Feature Extraction or Feature Selection

Spectral data are typically high-dimensional, strongly correlated, and often contain redundant information. Directly using such data for modeling can lead to overfitting, increased complexity, and reduced generalizability. Therefore, achieving dimensionality reduction through feature extraction or variable selection is vital for enhancing the model’s performance and interpretability [83].

Using mathematical projections, feature extraction transforms the original spectra into a lower-dimensional feature space. Common techniques include principal component analysis (PCA), independent component analysis (ICA), and recent nonlinear methods like t-distributed stochastic neighbor embedding (t-SNE) and Uniform Manifold Approximation and Projection (UMAP) [84,85]. PCA is most widely used, as it captures the main variance within the data and facilitates noise reduction and visualization. In contrast, variable selection directly identifies the most informative wavelengths from the original spectra, offering better physical interpretability. Typical approaches include the successive projections algorithm (SPA) [86], competitive adaptive reweighted sampling (CARS) [87], uninformative variable elimination (UVE) [88], and interval partial least squares [89] (iPLS). Recent trends favor integrated strategies combining both extraction and selection. For instance, hybrid approaches such as PCA and CARS or optimization-driven selection using genetic algorithms (GA) and particle swarm optimization (PSO) have been widely adopted [90]. Additionally, embedded selection methods based on Lasso, Elastic Net, or tree-based models like random forest (RF) and XGBoost are gaining traction in spectral analysis, as they can rank feature importance while fitting predictive models [91].

### 3.5. Model Calibration

The core goal of spectral data modeling is to effectively transform high-dimensional spectral signals into mathematical models that can predict target properties. In practice, modeling methods mainly fall into linear and nonlinear models. For systems with large sample sizes and relatively stable relationships between spectra and target properties, partial least squares regression (PLSR) remains the most mainstream approach due to its robustness, interpretability, and ability to handle multicollinearity [92]. Other commonly used linear models include multiple linear regression (MLR) and principal component regression (PCR), though they are generally less effective than PLSR when addressing multicollinearity and high-dimensional data. When there are pronounced nonlinear relationships between spectra and target properties, or when differences across sample batches increase the complexity of the modeling space, traditional linear models may become limited. In such cases, various nonlinear modeling approaches have been adopted. For example, support vector machines (SVM) enhance nonlinear fitting through kernel function mapping [93]. Random forest (RF) and gradient boosting decision trees (GBDT) use ensemble learning strategies [94], allowing them to capture complex interactions among variables and improve model robustness and generalization. Therefore, in practical applications, spectral data modeling often requires flexible adjustment of method selection based on sample characteristics, data structure, and the specific modeling task.

### 3.6. Model Evaluation

Model evaluation is an indispensable part of the spectral data modeling process, and the evaluation results directly influence model selection and optimization strategies. Common evaluation metrics can be categorized into goodness of fit, prediction error, and practical applicability. For regression tasks, goodness of fit is typically represented by the coefficient of determination (R^2^) or its equivalent correlation coefficient, which reflects the model’s ability to explain the variation in target properties within the calibration or test sets [95,96]. Prediction error metrics include the root mean square error of calibration (RMSEC) on the calibration set, the root mean square error of cross-validation (RMSECV), and the root mean square error of prediction (RMSEP) on independent test sets. These quantify prediction errors at different modeling stages. Practical applicability is often assessed using the residual predictive deviation (RPD). Generally, RPD > 2.5 indicates good practical performance, while RPD > 3.0 suggests strong applicability. For classification tasks, evaluation metrics include accuracy, sensitivity (recall), specificity, precision, and the area under the receiver operating characteristic curve (AUC–ROC). These indicators help measure the model’s ability to distinguish between different categories correctly, assess false positives and negatives, and evaluate overall classification reliability. Together, these metrics provide a comprehensive assessment framework, guiding the selection of suitable models and adjusting modeling strategies.

## 4. Application of Spectral Technology in Monitoring Mycotoxins

Grains and their derivatives are fundamental to human nutrition and social stability. However, they are prone to fungal contamination during storage, transportation, and processing. Traditional detection methods, while accurate, involve complex procedures and long turnaround times, making them less suitable for the rapid screening and batch monitoring required for bulk agricultural products. Vibrational spectroscopy has emerged as a promising alternative for mycotoxin detection, offering non-destructive, reagent-free, and online-compatible analysis. Driven by advances in spectral data representation, chemometric modeling, and predictive algorithms, research in this area has expanded rapidly, positioning vibrational spectroscopy as a key approach for non-invasive mycotoxin monitoring. This chapter reviews recent developments in applying vibrational spectroscopy to detect mycotoxins in grains and related products. It discusses spectral techniques, sample handling, wavelength coverage, feature extraction, and modeling strategies. Analyzing representative studies from 2019 to 2025 highlights the strengths and limitations of different approaches under specific grain and toxin scenarios, providing insights for overcoming current challenges and guiding future innovations.

### 4.1. Vis–NIR

Current research on applying Vis–NIR to mycotoxin detection mainly falls into two categories. One focuses on identifying contaminated versus normal samples, serving as qualitative screening. The other aims to build regression models that predict contamination levels, enabling semi-quantitative or quantitative analysis. Grains such as wheat, peanuts, and maize have received particular attention among various commodities. Vis–NIR has been utilized to monitor wheat quality throughout the production chain, from cultivation to processing and transport. For instance, Almoujahed et al. used Vis–NIR to detect Fusarium head blight in wheat. They acquired spectral reflectance data of wheat grains and flour within the Vis–NIR range and employed LDA and RF models to differentiate healthy from infected samples. Their approach was validated on both wheat kernels and flour. Results showed that an LDA model, optimized by recursive feature elimination, achieved 100% overall classification accuracy for both sample types, underscoring the feasibility of Vis–NIR for assessing potential toxin risks in processed products [97]. Building on this, Shen et al. developed an online Vis–NIR system to monitor wheat contamination by Fusarium and Aspergillus species, using a dataset of artificially inoculated samples [98]. By applying PCA combined with LDA, they achieved 91.7% accuracy in classifying different infection types and 88.3% in distinguishing infection levels (acceptable, mildly moldy, and heavily moldy). In another study, Tao et al. demonstrated the use of Vis–NIR to identify peanuts contaminated with aflatoxins [99]. They scanned peanut kernels with a Vis–NIR spectrometer covering 400–2500 nm, applied the Random Frog (RF) algorithm for feature selection, and built a PLS-DA model. Classification accuracies reached 90.0% and 94.29% at 20 ppb and 100 ppb AFB1 thresholds, respectively. These studies highlight the strong potential of Vis–NIR spectroscopy for early detection of fungal contamination in grain kernels.

Although Vis–NIR spectroscopy cannot directly capture characteristic absorption peaks of mycotoxins, it can reflect overall physicochemical changes in samples, such as differences arising from lipid oxidation, protein degradation, or color alterations during mold growth. Thus, when combined with appropriate modeling strategies and variable selection methods, Vis–NIR still holds the potential for predicting toxin content. Appaw et al. developed regression models in the 450–1050 nm wavelength range to analyze aflatoxin levels in white and yellow maize samples. According to their results, the models achieved cross-validation performance with R^2^ as high as 0.99, a limit of detection (LOD) of 0.60 ng/g, and a limit of quantification (LOQ) of 1.81 ng/g [100]. In addition, Almoujahed et al. constructed quantitative Vis–NIR models to predict DON contamination levels in wheat kernels and flour, comparing the effects of combining feature selection methods with ensemble learning algorithms [101]. When applying recursive feature elimination (RFE) to process the spectral data, the Extra Trees regressor yielded good prediction accuracy for DON in flour, reaching an R^2^ of 0.94. However, prediction performance for wheat kernels was less satisfactory, with an R^2^ of only 0.77 on the prediction set. Table 1 summarizes the application of Vis–NIR spectroscopy technology in mycotoxin detection in grains.

As summarized in Table 1, existing studies indicate that Vis–NIR has demonstrated initial practical potential for detecting mycotoxin contamination in grains and their derivatives. Although Vis–NIR cannot directly capture characteristic absorption peaks of toxin molecules, it excels at reflecting the macroscopic physicochemical states of samples, enabling indirect prediction of toxin concentrations. Current research has mainly focused on building classification and regression models for different grain matrices, such as wheat, maize, rice, and peanut meal. Among these, the PLS model remains the most widely applied tool, while some studies have introduced nonlinear algorithms like random forests to enhance model stability. Using variable selection methods helps improve model performance and computational efficiency; reducing redundant wavelengths and extracting effective features are key steps for boosting accuracy. In general, prediction performance has achieved R^2^ values around 0.90. However, model outcomes are still influenced by factors such as sample form (granular vs. powdered), matrix differences (including pigment interference), and the distribution of toxin concentrations. Despite these challenges, Vis–NIR spectroscopy combined with chemometric methods has become a promising candidate for non-destructive screening and rapid quantification of mycotoxins in grains.

### 4.2. NIR

Grains can be infected by various fungi belonging to the genera Aspergillus, Penicillium, and Fusarium. A primary concern is the production of toxins that pose significant health risks. Early identification and detection of such contaminants are critical control measures to ensure the safety of grains and grain-based products. Initial detection methods mainly relied on chromatographic and immunoassay-based high-throughput techniques. These approaches provide accurate insights into actual mycotoxin contamination levels in grains and offer excellent sensitivity and reliability. However, with the growing demand for large-scale on-site testing in recent years, their limitations have become increasingly evident, such as high costs and low efficiency. As a result, many researchers have sought greener and simpler detection techniques. NIR spectroscopy has gained considerable attention in recent years for mycotoxin detection and has become a prominent research focus. Numerous studies and reports continue to emerge on this topic, contributing to the further success of this approach. Table 2 summarizes the applications of NIR spectroscopy in detecting various mycotoxins in grains.

Compared to Vis–NIR, NIR spectroscopy has seen broader applications in grain analysis in recent years. It covers a wider range of mycotoxins and grain types and employs more strategic analytical approaches. Jiang et al. investigated the quantitative prediction of AFB1 produced by natural mold growth during wheat storage by comparing the performance of different feature selection methods combined with PLS models. When using 35 features selected by CARS to build the calibration model, they achieved the best detection accuracy, with an R^2^ of 0.9935 [105]. Another study explored the potential of using NIR spectroscopy to analyze ZEN contamination in wheat [106]. They employed a handheld NIR spectrometer to acquire spectra of ground wheat flour within the 900–1630 nm range and developed SVM models based on various feature selection methods. Results showed that the LASSO–SVM model significantly outperformed the others, achieving a correlation coefficient of 0.99 with only 28 selected features in the analysis. This demonstrates the capability of NIR to ensure wheat safety.

**Table 2:** The Application of NIR in the Detection of Mycotoxins.

Samples	Toxins	Range	Processing	Modeling	Performance	Ref.
Wheat	AFB1	4.1465–44.6981 ug/kg	Ground	PLSR	R^2^ = 0.9935	[105]
Peanut	AFB1	2.1207–290.0161 ug/kg	Ground	SVR	R^2^ = 0.9761	[107]
Maize	AFB1	2.6214–63.0195 ug/kg	Ground	PLSR, SVR	PLSR: R^2^ = 0.9260 SVR: R^2^ = 0.9707	[108]
Wheat	ZEN	19.6–65.0 ug/kg	Ground	SVR	R^2^ = 0.99	[106]
Wheat	ZEN	20.2380–483.9630 ug/kg	Ground	PLSR, SVR	PLSR: R^2^ = 0.9212 SVR: R^2^ = 0.9434	[109]
Maize	AFB1		Ground	BPNN	R = 0.9951	[110]
Maize	AF, AFB1	AF: 0.015–73.07 ug/kg AFB1: 0.015–30.17 ug/kg	Unground	PLS, ANN, PCA-DA	AF: R^2^ = 0.78, Acc = 100% AFB1: R^2^ = 0.82, Acc = 97.4%	[111]
Black beans	Fumonisin B1	0–10 mg/kg	Unground/ Ground	PLSR	R^2^ = 0.92	[112]
Peanut	AFB1	2.21–23.79 g/kg	Unground	LDA, PLSR	R^2^ = 0.942, Acc = 100%	[113]
Peanut	AFB1	0.63–56.0 ug/kg	Ground	CNN	R^2^ = 0.99	[114]
Rice	AFB1	0–41.990 ug/kg	Ground	PLS-DA, PLSR	R = 0.952, Acc = 90%	[115]
Maize	FB1, FB2	FB1: 62.5–4000 ug/kg FB2: 62.5–2861 ug/kg	Ground	PLSR, ANN	FB1: R^2^ = 0.91 FB2: R^2^ = 0.93	[116]
Barley	Enniatin	5.4–7459.2 ug/kg	Ground	PLS-DA	Sensitivity = 94.2%	[117]
Peanut	AFB1	2.44–223.76 ug/kg	Unground	Naïve Bayes	Acc = 86.96%	[118]
Maize	FB1, FB2	0–217.45 mg/kg	Ground	PLSR, SVR, LPLS-S, PLS-DA, SVM-DA	R^2^ = 0.91, Acc = 89.3%	[119]
Maize	AFB1	2.6214–63.0195 ug/kg	Ground	CNN	R^2^ = 0.9955	[120]

The quality of peanut products can also benefit from advances in NIR spectroscopy. One of the earliest studies confirmed this possibility [118]. In their work, individual peanuts were deliberately inoculated with Aspergillus flavus strains, producing samples with AFB1 concentrations ranging from 2.44 to 223.76 μg/kg. NIR spectra were collected in the 940–1660 nm range. A naïve Bayes classifier was developed using full-spectrum data and data optimized by the SPA. Results showed that the SPA-based classifier achieved the best classification accuracy of 91%, successfully distinguishing normal samples at a threshold of 20 μg/kg. Research on monitoring mycotoxin contamination in peanuts using NIR has continued since. In the following year, Yao et al. further validated the feasibility of this approach [113]. They employed FT-NIR spectroscopy to acquire data from two sampling modes on peanut kernels, reflection and transmission, and developed quantitative (PLSR) and qualitative (LDA) models. After applying different preprocessing methods combined with PCA, both modeling approaches perfectly discriminated between normal and contaminated samples. Quantitative results also provided strong evidence, with PLS models built on reflection and transmission data achieving prediction accuracies of 0.998 and 0.917, respectively, on external validation sets. Li et al. recently proposed a composite optimization strategy that accurately predicted AFB1 levels in peanuts using an SVM model and explored a broader detection range [107]. They first performed a standard NIR analysis workflow, including preprocessing and feature selection. Then, they employed an intelligent optimization algorithm (beluga whale optimization, BWO) to find parameters that would maximize model accuracy. Ultimately, with 18 selected variables as input, the model achieved a prediction performance of 0.9761.

Existing studies indicate that NIR spectroscopy and FT-NIR have demonstrated feasibility for non-destructive quantitative detection of mycotoxins in grains. Models built on conventional NIR regions (approximately 900–2500 nm) and FT-NIR high-resolution ranges (such as 10,000–4000 cm^−1^) generally achieve R^2^ values exceeding 0.92. This suggests that spectral systems respond well to indirect chemical changes associated with toxin contamination, such as lipid oxidation and protein degradation. Moreover, combining appropriate chemometric and data analysis methods can improve detection accuracy, as shown in studies [114,120]. In these investigations, one-dimensional NIR spectral curves were transformed into two-dimensional images, enhancing feature clarity. Calibration models developed by mining these images with convolutional neural networks achieved stable detection accuracy around 0.99. This highlights the advantages of deep learning approaches in NIR spectral analysis. It is also noteworthy that although some studies summarized in the tables involved spectral acquisition on whole grain kernels, most reported experiments ground the grains to obtain more uniform samples. In practice, powdered samples generally yield higher model fits due to improved spectral transmission and homogeneity. However, this also poses a major obstacle to on-site applications.

### 4.3. MIR

MIR spectroscopy features a unique fingerprint region, which gives most compounds distinctive spectral characteristics in this range. In other words, it is uncommon for different compounds to display identical spectral profiles here. This advantage makes MIR spectroscopy suitable for both qualitative and quantitative analyses. As modern technologies have advanced, including chemometrics, computing, and hardware improvements, the potential of MIR spectroscopy for non-destructive detection has gradually attracted increasing attention from researchers. The earliest studies attempting to use MIR spectroscopy for detecting fungal infections or mycotoxins in various samples date back to 2001. In the years that followed, many researchers began to explore the capability of this technique to detect fungal contamination specifically in grains. Table 3 lists several representative studies identified within the time range covered by the references in this work.

Wheat and its derived products are highly affected by Fusarium head blight (FHB), which can lead to the production of DON when the disease becomes severe. MIR spectroscopy has already been applied for the early screening of FHB in wheat. Almoujahed et al. compared the ability of MIR spectroscopy to detect FHB in whole wheat kernels and wheat flour [97]. They collected MIR spectral data in the 400–1700 nm range and used these data to develop RF and LDA classification models. Results indicated that MIR spectroscopy performed better in powdered wheat flour, achieving 100% classification accuracy. For whole kernels, detection was also acceptable, with an accuracy of 93.1%. A recent study extended this work by successfully screening normal and abnormal wheat samples according to European Union (EU) standards and analyzing DON contamination using attenuated total reflectance MIR spectroscopy [121]. Their developed PLS-DA model achieved an average true positive rate of 81% for identifying normal samples. In addition, OTA contamination in wheat can also be assessed using MIR spectroscopy. This was demonstrated by Girolamo et al. [122], who collected 229 naturally OTA-contaminated wheat samples and acquired data with FT-MIR spectroscopy. They evaluated the feasibility of the technique by developing PCA-LDA and PLS-DA models. Results showed that individual wheat samples could be identified at rates exceeding 94% even at the lowest concentration of 2 μg/kg. This demonstrates the potential of this technology, given that the minimum acceptable limit for OTA in unprocessed wheat is 5 μg/kg according to EU and Chinese regulations. In the same year, another study further expanded the application of MIR spectroscopy in screening mycotoxins in grains [123]. They proposed using MIR spectroscopy to identify peanut kernels free from aflatoxin contamination. By artificially contaminating normal peanuts, they obtained samples with concentrations ranging from 3.24 to 2951 ppb and collected the corresponding spectral data. They then developed discrimination models such as orthogonal partial least squares discriminant analysis (OPLS-DA) and soft independent modeling of class averages (SIMCA), along with a quantitative detection model based on PLSR. The results demonstrated the promise of this technique. Both classification models achieved over 94% accuracy when aflatoxin levels exceeded 3 ppb, and the developed PLSR model reached a correlation coefficient of 0.85 for quantifying toxin content in single kernels, with detection completed in about one minute. This highlighted the rapid and non-destructive nature of the method.

**Table 3:** The application of MIR in the detection of mycotoxins.

Samples	Toxins	Range	Processing	Modeling	Performance	Ref.
Wheat	FBH	/	Unground/ Ground	RF, LDA	Kernels: Acc = 93.1% Flour: Acc = 100%	[97]
Wheat	DON	/	Ground	PLS-DA	Balanced dataset: true positive rate (TPR): 0.81 Imbalanced dataset: true negative rate (TNR): 0.85	[121]
Peanut	AF	3.24–2951.21 ppb	Unground	OPLS-DA, SIMCA, PLSR	R = 0.85, Sensitivity = 94.7%	[123]
Wheat	OTA	0.15–54 ug/kg	Unground	PLS-DA, PC-LDA	Acc = 96%	[122]

These studies have fully demonstrated the core potential of MIR spectroscopy, particularly in its attenuated total reflectance (ATR) sampling mode, for rapid screening of mycotoxins in grains. This technique can directly perform non-destructive and fast detection on whole, unground grain kernels, greatly simplifying sample preparation and aligning more closely with the practical needs of on-site rapid screening. However, it is important to clarify the current reality. Compared to the overall surge of literature on toxin rapid detection using vibrational spectroscopy, especially NIR, studies based on MIR technology remain relatively scarce. This striking contrast is likely due to the extreme sensitivity of MIR to the high moisture content in grain matrices and its shallow penetration depth, which poses challenges for samples with uneven toxin distribution. Therefore, future research should place greater emphasis on addressing this phenomenon.

### 4.4. Raman

There is growing awareness of the health benefits associated with the quality of grain products. With the rapid increase in the global population, the demand for grain-based food is no longer considered solely from a quantity perspective. Reducing grain losses has become an important way to alleviate food shortages, particularly by minimizing fungal contamination along the consumption chain. A significant portion of global food waste results from improper storage and transportation, which leads to fungal contamination and renders the grains unusable. As a member of the vibrational spectroscopy family, Raman spectroscopy has gradually been applied to detecting mycotoxins in grains and their products in recent years. Table 4 summarizes several studies that have employed Raman spectroscopy to detect grain toxins.

At the early stages of research, most work employed standard Raman spectroscopy combined with chemometric methods to achieve detection objectives. An initial study investigated the potential of Raman spectroscopy for detecting ZEN in maize [128]. Using a 785 nm laser, they scanned maize samples with ZEN concentrations ranging from 6.9 to 800 μg/kg. Important spectral features were selected through a genetic algorithm and synergy interval partial least squares (SiPLS). This resulted in an optimal PLSR detection model built on only 0.39% of the full-spectrum variables, achieving a R 0.9260. This study demonstrated the feasibility of applying Raman spectroscopy to detect grain toxins. In the following years, several other publications reported similar work. For example, handheld Raman spectrometers were used to detect AFB1 and ZEN in maize, wheat, and peanuts [124,126,127]. Although the toxin ranges investigated were narrower, generally between 2 and 300 μg/kg, compared to [128], they reflected real-world scenarios of fungal contamination. Grains already show visible alterations at very high concentrations and are beyond salvage. These studies further confirmed that Raman spectroscopy can be effectively utilized for this purpose. Detection accuracies typically ranged from 0.95 to 0.99, with most studies incorporating feature optimization techniques. They also emphasized that feature optimization or extraction had a significant positive impact on modeling performance.

The above studies indicate that Raman spectroscopy, when combined with appropriate chemometric methods, can be used to detect specific concentrations of mycotoxins in grains. However, due to the inherently weak Raman signals, the detectable toxin thresholds are relatively high. As a result, surface-enhanced Raman spectroscopy (SERS) has become more widely accepted in practical applications. This is further evidenced by the growing number of publications on SERS in recent years. This increasing trend is closely tied to advances in nanotechnology, with nanomaterials naturally becoming one of the core elements in developing SERS since they are essential for signal enhancement. Based on this foundation, the primary focus of SERS research has been optimizing nanostructured substrates and innovating probe designs, enabling the technique to achieve high specificity, a broad linear range, and ultra-high sensitivity in mycotoxin detection. Table 5 summarizes several SERS applications using various substrates to detect grain mycotoxins. While the listed studies do not represent a complete compilation, they are sufficient to illustrate the current development directions.

Compared to conventional Raman spectroscopy, achieving extremely low detection limits is a major goal of applying SERS, typically reaching trace levels or even lower. For example, Yin et al. proposed a SERS method for detecting ZEN in maize [130]. They used gold nanostars (AuNSs) as capture probes and linked them with core–shell structures (AuMBA@AgMBANPs). The coupling between AuNSs and AuMBA@AgMBANPs created electromagnetic fields that amplified the Raman signals. This approach established a robust linear relationship in the range of 5 to 400 μg/kg (R^2^ = 0.9989). When applied to real maize samples, the method achieved recoveries between 90.58% and 105.29%, with an LOD of 3 μg/kg, showing good agreement with HPLC-FLD methods. Clearly, this satisfies the regulatory minimum limits of 60 μg/kg. The same team further improved this detection limit in another study [131]. They developed a novel SERS aptasensor using mesoporous silica loaded with gold nanoparticles (Rh6G–MSN–AuNPs) as the enhancement substrate, with aptamers serving as specific recognition and masking probes. The tiny nanogaps between AuNPs allowed MSN–Rh6G–AuNPs to exhibit strong SERS performance under laser excitation. This sensor demonstrated a good linear range from 3 to 200 ng/mL and achieved an LOD of 0.0064 ng/mL (0.0064 μg/kg). In addition, SERS techniques targeting FB1, AFB1, and OTA in wheat and peanuts are also under continuous development. Gao et al. proposed a dual-mode Raman/fluorescence aptasensor for detecting AFB1 in peanuts [132]. This sensor was assembled from gold nanoparticles (AuNPs) and magnetic nanoparticles (MNPs) and could simultaneously achieve SERS and fluorescence resonance energy transfer (FRET) effects. Spike experiments in peanut samples validated its practicality, yielding recoveries from 91.4% to 95.6% with a detection limit as low as 5.81 pg/mL. In recent years, as this technology has matured and gained increasing attention, researchers have moved beyond laboratory conditions and focused on enhancing its practical usability for on-site testing. As a result, rapid detection products based on this technology have emerged. For example, Yin et al. developed a SERS-based test strip for detecting ZEN in maize [133]. The strip used core–shell Au@AgNPs embedded with reporter molecules (4-MBA) as SERS nanotags. Final results demonstrated that this test strip could detect ZEN in maize samples across a range of 10 to 1000 μg/kg, with an LOD of 3.6 μg/kg, and the SERS analysis time was less than 20 min. Combined with portable and handheld Raman spectrometers, this technology shows promise as a powerful tool for field detection of mycotoxins.

**Table 5:** The Application of SERS in the Detection of Mycotoxins.

Samples	Toxins	Range	SERS Substrate	Performance	LOD	Ref.
Wheat Maize	OTA ZEN	OTA: 0.01–100 ng/mL ZEN: 0.05–500 ng/mL	Reporting probe: Au@Ag core–shell nanoparticles modified 4-MBA and DTNB Capture probe: Gold nanorods (AuNRs) modified complementary DNA (SH-cDNA)	OTA: R^2^ = 0.986 ZEN: R^2^ = 0.987	OTA: 0.018 ng/mL ZEN: 0.054 ng/mL	[134]
Maize	ZEN	5–400 μg/kg	AuMBA@AgMBANPs	R^2^ = 0.9989	3 μg/kg	[130]
Maize	ZEN	3–200 ng/mL	MSN–Rh6g–AuNPs	R^2^ = 0.988	0.0064 ng/mL	[131]
Maize	ZEN	10–1000 μg/kg	Core–shell Au@AgNPs with embedded reporter molecules (4-MBA)	R^2^ = 0.993	3.6 μg/kg	[133]
Wheat	AFB1	0.1–5 ng/mL	Au@Ag core–shell nanoparticle (Au@Ag CSNPs)	R^2^ = 0.9963	0.03 ng/mL	[135]
Coix seed	AFB1	0.01–100 ng/mL	gold magnetic nanoparticles (GMNPs) and Ag NPs	R^2^ = 0.9948	0.0060 ng/mL	[136]
Wheat	FB1	0.01–1 µg/L	Se-WCDs-Au-Janus Ag NPs	R^2^ = 0.9883	0.005 μg/L	[137]
Peanut	AFB1	0.01–100 ng/mL	AuNPs and MNPs	R^2^ = 0.9742	5.81 pg/mL	[132]

In summary, Raman spectroscopy can be a powerful tool for screening mycotoxins in grains. However, traditional Raman spectroscopy is still affected by weak signals and grain matrix variations. SERS technology alleviates the issue of high detection limits in traditional Raman spectroscopy, but the application of this technology in practical on-site detection still requires further attention.

### 4.5. HSI

Unlike traditional spectroscopic techniques such as NIR and Raman mentioned earlier, HSI collects both spectral and spatial image information of samples, drawing upon conventional computer vision approaches. This capability allows HSI to capture multidimensional features of the samples. As a result, HSI can more reliably represent the true condition of target samples. HSI has already been developed for detecting various mycotoxins in different grains, as shown in Table 6.

Several studies have first reported promising results using HSI to classify wheat and maize samples contaminated by Fusarium or DON. For example, Femenias et al. used an 895–1728 nm NIR-HSI system to measure DON content in individual wheat kernels that were artificially contaminated [138]. They developed LDA, Naïve Bayes, K-NN, and ANN models to distinguish whether wheat was infected by Fusarium and to determine if DON levels exceeded the limits set by EU regulations. Their results showed differences in HSI spectral profiles among infected kernels, DON-contaminated kernels, and uncontaminated kernels, particularly in the 1100–1400 nm range. Using ANN models developed on absorbance-transformed spectra yielded the best performance for identifying whether wheat was infected by Fusarium (Acc = 85.8%). For determining whether DON levels exceeded EU thresholds, the best detection model was based on SNV-pretreated data combined with ANN (Acc = 76.9%). Additionally, a quantitative model developed using PLS achieved a detection accuracy of R^2^ = 0.88, indicating that NIR-HSI is suitable for predicting DON content in individual wheat kernels. Shen et al. employed an NIR-HSI system (900–1700 nm) to classify and quantify the contamination levels of naturally DON-contaminated field wheat kernels [139]. They collected spectral data from two regions of each wheat kernel, the ventral and dorsal sides. Results showed that in a three-class classification task for contamination levels, a PLS-DA model developed using spectra acquired from the dorsal side achieved 100% accuracy in correctly classifying all wheat samples. Although the LPLS-S model achieved the best quantitative performance with an R^2^ of 0.81, it required combining spectral data from both the dorsal and ventral sides. Nevertheless, this study highlighted the effectiveness of the NIR-HSI technique. Several similar studies have also been conducted on wheat-derived products. Zhao et al. used Vis–NIR hyperspectral imaging to evaluate the feasibility of detecting DON contamination in whole wheat flour [140]. They collected 195 wheat flour samples from 13 cities to incorporate batch heterogeneity into the study. Spectral data, color features, and texture features were acquired, and PCA, LDA, and PLS were applied to classify and quantify DON content. Results showed that when the classification threshold was set at 1 mg/kg, a PCA-LDA model developed on fused spectral and color features achieved good classification performance with an accuracy of 96.92 percent. However, the quantitative analysis revealed an opposite conclusion. The technique failed to meet quantitative detection requirements within the range of 0.0 to 6.233 mg/kg, yielding a correlation coefficient of only 0.691. This limitation might be related to the chemometric methods and selections employed, since a more recent study under similar conditions reported satisfactory results. Saini et al. employed a close-range HSI system covering 397–1004 nm to quantify DON levels ranging from 0.9 to 57.2 mg/kg in wheat flour [141]. They collected 241 wheat kernel samples, which were then ground using a milling device to obtain powdered wheat flour samples. The study compared the effects of different data forms and chemometric methods, specifically contrasting machine learning with deep learning approaches, on detection performance. Particular attention was given to evaluating 1D-CNN, 2D-CNN, and 3D-CNN models combined with data augmentation strategies. The 1D-CNN used spectral features as input, and the 2D-CNN took two-dimensional images of wheat flour. At the same time, the 3D-CNN employed the full hyperspectral data cubes across 397–1004 nm, representing the most direct data format from the HSI system. Results showed that data augmentation significantly improved model performance, a trend consistently observed across all models. This effect was especially pronounced in the 1D-CNN, where the model’s performance increased from 0.90 to 0.96, achieving the best detection accuracy in the study. These findings indicate that the choice of chemometric or modeling methods positively impacts enhancing HSI detection performance.

**Table 6:** The Application of HSI in the Detection of Mycotoxins.

Samples	Toxins	Range	Processing	Data Type	Modeling	Performance	Ref.
Wheat	DON	0.9–57 mg/kg	Ground	Spectral value	PLSR, RF, SVR, CNN	R^2^ = 0.96	[141]
Maize	AFB1	0–1206 ug/kg	Ground	Spectral value	LDA, SVM, QDA	Vis–NIR: Acc = 82.6% Fluorescence: Acc = 95.7% SWIR: Acc = 95.7% Raman: Acc = 87%	[142]
Wheat	DON	0–135.7 mg/kg	Unground	Spectral value	LDA, Naïve Bayes, KNN, ANN, PLSR	R^2^ = 0.88, Acc = 76.9%	[138]
Wheat	DON	<LOD−6.233 mg/kg	Ground	Spectral features, Image features	LDA, PLSR	R = 0.691, Acc = 96.92%	[140]
Wheat	DON	<LOD − 2.7 mg/kg	Unground	Spectral features	LDA	Acc = 92.5%	[143]
Wheat	DON Fusarium	/	Unground	Spectral features, Image features	KNN	DON: Acc = 80%, Fusarium: Acc = 85%	[144]
Wheat	DON	<LOD−507.28 mg/kg	Unground	Spectral feature	PLS-DA, PLS, SVM, LPLS-S,	Acc = 100%, R^2^ = 0.81	[139]
Peanut	AFB1, AFB2, AFG2, AF	AFB1: 0.148–84.038 AFB2: 0.011–73.625 AFG2: 0–9.163 AF: 0.159–166.826	Unground	Spectral feature	PLS-DA, LDA, SIMCA, K-NN PLSR, PCR	AFB1: R^2^ = 0.8863, Acc = 89.66% AFB2: R^2^ = 0.7864 AFG2: R^2^ = 0.6612 AF: R^2^ = 0.8559, Acc = 79.31%	[145]
Maize Kernels	DON, FB1, FB2, FB1 + FB2	DON: <LOD−18.622 FB1: <LOD−37.591 FB2: <LOD−27.066 FB1 + FB2: <LOD − 63.891	Unground	Spectral feature	RF, ANN, KNN, logistic regression, PLSR	DON: Acc = 98.60% FB1 + FB2: Acc = 84.40% DON+ FB1 + FB2: Acc = 89.8% DON: R = 0.904 FB1: R = 0.868 FB2: R = 0.901 FB1 + FB2: R = 0.901	[146]
Oat	DON	<LOD-2706 µg/kg	Unground/ Ground	Spectral feature	RF, ANN, KNN Naïve Bayes, PLSR	Unground: Acc = 77.8% Ground: Acc = 70.8% Unground: R = 0.92 Ground: R = 0.90	[147]
Peanut	AFB1	0–200 ppb	Unground	Spectral feature Texture feature Color feature	LDA, PLS-DA, SVM	Acc = 94%	[148]
Maize	AF	LOD−>2000 ppb	Unground	Spectral feature	PLS-DA	20 ppb: Acc = 89.8% 100 ppb: Acc = 89.3%	[149]
Maize	AF	LOD−>2000 ppb	Unground	Spectral feature	LDA	20 ppb: Acc = 86.7% 100 ppb: Acc = 89.6%	
Maize	AF FM	/	Unground	Spectral feature	PLS-DA, SVM	Vis–NIR: Acc = 89.1% Fluorescence: Acc = 71.7% SWIR: Acc = 95.7%	[150]
Maize	ZEN	19.98–102.30 µg/kg	Unground	Spectral feature	BPNN, PLS-DA, SVM	R^2^ = 0.95, Acc = 93.33%	[151]
Peanut	AF	/	Unground	Spectral feature	PLS-DA, PCA-LDA, LDA, ISOGA-CNN, CNN, CNN-LSTM, A-CNN-LSTM	Binary: Acc = 93.33% Six: Acc = 100%	[152]

In fact, beyond these studies, research on applying HSI to detect mycotoxins in grains is being explored in greater depth and is no longer limited to a single type of grain or toxin. As demonstrated in Table 6, HSI is receiving increasing research attention as a particular analytical technique. Its feasibility has already been demonstrated for detecting aflatoxins and fusarium mycotoxins (FB1 and FB2) in grain matrices such as peanuts, maize, and oats [146,147,153]. However, whether focusing on the degree of fungal contamination or the quantification of toxins, the stability of HSI performance across different grain matrices still requires close attention. For classification tasks, detection accuracies have ranged from 70 to 100 percent, with most reported results clustering around 90 percent. Although this classification level meets general requirements for chemical analysis, from the perspective of broader applicability, there remains room for improvement. Regarding quantification, most models based on HSI have achieved detection accuracies with R^2^ values around 0.90, yet some models still fail to meet analytical standards. Given that HSI systems typically do not require grinding samples, these outcomes seem reasonable because fungal contamination is not evenly distributed within kernels. On the other hand, many existing studies rely solely on extracted spectral data without integrating the visual aspects of the information. This is likely linked to computational constraints, since processing full three-dimensional hyperspectral cube data demands substantial computational resources. This challenge may also be one of the key obstacles to the broader application of the technique.

## 5. Difference Analysis of Different Vibrational Spectroscopy Techniques

Vibrational spectroscopy techniques have been applied to varying extents for detecting mycotoxins in grains, with each technique playing its distinct role. However, there are still significant differences among them regarding fundamental principles, spectral information acquisition capabilities, performance characteristics, suitability, and operational requirements. Based on the studies reviewed in this work, Table 7 summarizes several key aspects and highlights the application features of each technique.

Firstly, from the perspective of information acquisition, these techniques provide chemical fingerprints at different levels. MIR spectroscopy directly detects fundamental vibrations of molecular groups in the “fingerprint region”. It offers the highest level of molecular specificity. This allows it to distinguish different chemical bonds and functional groups clearly. MIR is highly valuable for directly identifying target mycotoxins and confirming their structures in complex matrices. Raman spectroscopy also provides molecular vibration information. It is based on an inelastic scattering mechanism. Its main advantage is that it is not sensitive to water. This makes it suitable for directly analyzing high-moisture or liquid foods. However, regular Raman signals are weak. This limits its sensitivity. SERS addresses this by using local surface plasmon resonance from nanometal structures. It can amplify Raman signals by millions of times. As a result, it achieves detection at trace or ultra-trace levels (ng/kg to pg/kg). This is hard for other vibrational spectroscopy techniques to reach. Vis–NIR and NIR spectroscopy mainly capture overtones and combination bands of O-H, C-H, and N-H groups. Vis–NIR also records color information. These techniques reflect overall physicochemical states, such as moisture, fat, protein content, and color changes. Because of strong spectral overlaps, their specificity is lower. Detecting toxins depends on the indirect changes caused by contamination. Powerful chemometric models are used to build these correlations. HSI technology, on the other hand, is unique in that it combines spectral analysis and imaging techniques to acquire spectral information at each pixel point and provide an image of the spatial distribution of the sample. This allows HSI to visualize the heterogeneous distribution of mycotoxins or mold regions in food samples (especially whole grains), which is impossible with point-of-view techniques.

These fundamental differences directly lead to their advantages and disadvantages in key performance metrics. In terms of sensitivity (LOD), SERS, with its powerful signal enhancement capability, is far ahead. As shown in the studies in Table 5, it generally reaches ng/kg or even pg/kg levels, such as 0.0064 ng/mL ZEN reported in [131], making it the first choice for trace single-target detection. MIR and Raman, relying on their ability to acquire molecular information directly, usually achieve μg/kg levels. NIR and Vis–NIR also mostly stay within the μg/kg range, but their sensitivity depends more on the model and sample state, such as whole grains versus powders. The LOD of HSI is generally similar to or slightly higher than that of NIR. Regarding selectivity or specificity, the fingerprint region of MIR gives it the highest inherent selectivity. The selectivity of SERS can be significantly improved by designing specific recognition elements, such as aptamers or antibody-functionalized substrates. However, the selectivity of Raman, NIR, Vis–NIR, and HSI is relatively lower. They mainly rely on subsequent data analysis and variable selection techniques to distinguish target signals from background interference.

Sample pretreatment requirements and applicability are key factors in determining whether a technique suits on-site or online applications. Vis–NIR, NIR, and HSI have clear advantages in non-destructive testing. They are especially well-suited for rapid screening of solid samples such as whole grains and nuts, as shown by the numerous studies on intact samples in Table 1, Table 2 and Table 6. HSI is particularly adept at handling samples with spatial heterogeneity and does not require homogenization. Raman is insensitive to moisture and can directly analyze moist samples’ liquids and surfaces. It is also relatively feasible for intact grain testing. MIR, however, is limited by its shallow penetration depth and extreme sensitivity to moisture, since water has strong absorption peaks. It usually requires complex sample pretreatment, such as drying, grinding into powders, making KBr pellets, or using an ATR accessory for contact measurements on solid surfaces. These needs restrict its application to detect whole, unprocessed grain samples rapidly. Although SERS offers extremely high sensitivity, its detection process often involves toxin extraction or requires the sample to be brought into contact with or incubated on specific nano-enhanced substrates. This remains the main obstacle to achieving true non-destructive, in situ detection, although portable SERS detection strips, such as those in [133], are working to address this issue.

Overall, no single vibrational spectroscopy technique serves as a “universal key” for all mycotoxin detection scenarios. Vis–NIR, NIR, and FT-NIR, with their speed, non-destructive nature, and suitability for online and high-throughput applications, are ideal for large-scale rapid screening of grains and overall quality monitoring. MIR stands out for its high specificity and offers unique value for toxin confirmation, molecular structure studies, or analyses of homogeneous samples in laboratory settings. The strength of Raman lies in directly analyzing moist samples and performing non-destructive surface analysis. SERS demonstrates unmatched potential when extreme sensitivity is required for trace-level and highly specific target toxin detection, though its operational complexity and cost remain challenges to be addressed. The core advantage of HSI is its ability to provide visualized spatial distribution information of toxins or mold. This makes it a powerful tool for studying contamination heterogeneity, locating moldy regions, and achieving non-destructive grading of whole samples. Selecting the most appropriate technique must take into account multiple factors, including the type of target toxin and regulatory limits, the characteristics of the food matrix (solid or liquid, moisture content, homogeneity), as well as requirements for detection speed, throughput, cost, and whether non-destructive analysis or spatial information is needed.

## 6. Challenges, Trends and Outlook

Vibrational spectroscopy is an indirect detection technique that obtains sample information based on the interaction between molecules and electromagnetic radiation. It is known for requiring little or no sample preparation, providing rapid results, offering good cost efficiency, and being non-destructive. Monitoring grain quality, especially regarding mycotoxin contamination, has greatly benefited from this technology. However, future work still needs to address challenges and limitations to improve its applicability further.

1. The trend of further development in chemometrics. Chemometrics, including pattern recognition and regression analysis methods, serves as a bridge for linking spectral features with target properties. Although each spectroscopic technique carries different levels of features that can be used to assess chemical and structural information, extracting, utilizing, and interpreting the hidden information and patterns within these data remain major challenges. This is especially true when dealing with low levels of mycotoxins in grains, where spectral response signals are weak and exhibit strong nonlinearity. Efficiently mining key information related to contamination from complex spectral data becomes a decisive factor for model performance. Many studies have reported that the combined application of chemometric strategies can push the limits of spectral analysis [154]. At present, widely used regression modeling processes, such as partial PLSR or SVR, do offer specific modeling capabilities. However, their construction relies on manual feature selection and stepwise optimization, which makes it difficult to fully capture deep relationships among high-dimensional, nonlinear, and redundant features. Moreover, these methods often assume independence among feature variables, ignoring possible synergistic coupling structures across spectral channels, limiting models’ generalization ability. Therefore, future chemometric analysis should gradually evolve toward deep modeling approaches driven by artificial intelligence and big data. In particular, end-to-end feature learning architectures such as CNN, residual networks (ResNet), and Transformers can automatically perform feature extraction, representation compression, and nonlinear modeling without explicit preprocessing or variable selection. They can discover weak feature signals through end-to-end learning. Such applications have already begun to emerge [155].

2. The necessity of developing standardized and shared spectral databases. Research on applying vibrational spectroscopy for mycotoxin detection in grains is advancing rapidly. However, there are still common problems such as a lack of sample data resources and insufficient model generalization ability. The types of mycotoxin contamination in grains are highly complex. More than 400 types are known, with diverse sources and formation mechanisms. Grains include various crops such as maize, wheat, barley, peanuts, and rice. Each matrix differs significantly in moisture content, particle size, color, and tissue structure. Additionally, because mycotoxin contamination is often hidden and heterogeneous, manual sampling and reference value determination (such as by HPLC or ELISA) are time-consuming and labor-intensive. This severely restricts large-scale sample acquisition, further intensifying the scarcity of modeling data. Spectral modeling heavily relies on representative samples. Small datasets from a single laboratory cannot adequately reflect real-world situations, especially when facing multiple grain types, toxin varieties, and contamination levels. Therefore, it is urgent to build large, standardized spectral databases that cover different matrices, toxin types, and contamination levels. More importantly, models constructed from shared databases could support model transfer, domain adaptation, method evaluation, and visualization interpretation in the future. This would significantly shorten the development cycle and reduce the validation costs of new applications. Furthermore, the ability to maintain and transfer models needs to be improved. Vibrational spectroscopy combined with chemometrics has already achieved many modeling results in mycotoxin detection. However, its practical application is still constrained by a core problem: strong model specificity but weak generalization. Current modeling strategies build local models for specific toxins, matrices, and instruments. This means that adding a new toxin type, a new grain matrix, or even a new batch of samples or instruments often requires retraining or model optimization. Such requirements greatly limit model universality and application efficiency. Future spectral analysis should focus on developing adaptive learning frameworks. By using transfer learning, pretrained model knowledge can be migrated to new scenarios. Domain adaptation techniques can reduce the data distribution gap between source domains (laboratories) and target domains (field sites). At the same time, incremental learning algorithms should be developed so that models can continuously incorporate new sample data without complete retraining. Online calibration modules should complement this to compensate for real-time instrument drift and ensure long-term model robustness. In fact, research on transfer learning has already begun [156].

3. Multispectral synergistic techniques can provide more comprehensive analyses. Each spectroscopic technique has its unique characteristics. NIR is sensitive to overall component changes but has weak specificity. MIR offers molecular fingerprint specificity yet is affected by moisture interference. HSI provides spatial distribution information but generates massive amounts of data. These techniques have clear advantages but also exhibit detection blind spots. As a result, models built on a single modality often encounter performance bottlenecks when sample complexity increases or contamination heterogeneity becomes pronounced. In the future, multispectral synergistic integration is expected to become a key direction for improving analytical accuracy and enhancing detection dimensions. By integrating data collected from different spectroscopic techniques, it is possible to preserve the original feature advantages while achieving enhanced expression of complementary information. For example, HSI can first be used to locate moldy areas, followed by MIR micro-area sampling for precise toxin identification. NIR can rapidly perform initial screening, combined with Raman spectroscopy for molecular confirmation of suspicious samples. This kind of synergy can greatly improve the resolution of complex contamination problems.

4. Instrument miniaturization and portability are key to achieving on-site applications. Vibrational spectroscopy has achieved many results in laboratory settings. However, deploying this technology in on-site grain production, storage, and processing still faces significant challenges. Large equipment size, complex structures, and reliance on stable environmental conditions limit portability and field applications. This is especially critical in detecting mycotoxins in grains, where contamination often shows high spatial heterogeneity and dynamic temporal changes. The response is delayed and inefficient if relying on laboratory analysis, which can easily miss early identification windows. With advances in optical device integration and miniaturization technologies, portable and handheld spectrometers have continued to emerge. Some miniature near-infrared devices even support connections with smart terminals, enabling “plug-and-play” functions that allow data collection and analysis simultaneously. However, these portable devices still face limitations in detection performance, low signal-to-noise ratios, and poor stability. This is particularly evident when dealing with trace-level toxins or complex matrices, where detection sensitivity and accuracy still lag behind laboratory-grade instruments. Future portable vibrational spectroscopy devices will need continuous hardware improvements in light sources, detectors, signal enhancement, and noise suppression.

5. The distance from laboratory to industrial deployment still needs to be narrowed. Laboratory validation of spectroscopic techniques primarily focuses on achieving high accuracy and precision in controlled environments. In this setting, systems are typically designed for low-throughput analysis, where high-resolution instruments and complex preprocessing steps are employed to optimize detection performance. The goal is to develop robust models that identify mycotoxins with minimal interference, often emphasizing experimental control, fine-tuning, and reproducibility. However, these systems are often costly, require skilled operators, and are not designed for continuous or real-time use in industrial environments. In contrast, industrial deployment of spectroscopic systems prioritizes cost-effectiveness, throughput, and ease of use. For large-scale grain storage and processing lines, equipment must be durable, capable of high-throughput analysis, and able to operate in variable environmental conditions with minimal downtime. The systems must also be user-friendly, requiring little to no specialized operator training. This presents a challenge, as most high-performance laboratory systems are expensive and may not be suitable for mass-scale, rapid, and real-time monitoring. Additionally, while laboratory models are often validated under controlled conditions, industrial applications require regulatory validation to ensure compliance with food safety standards, including developing standardized protocols and performance benchmarks. As a result, bridging the gap between laboratory methods and industrial applications requires both technological adaptation and the creation of standardized procedures that ensure both performance and regulatory compliance.

6. Moisture sensitivity and limited penetration depth are key challenges for vibrational spectroscopy in grain storage and processing. The susceptibility of vibrational spectroscopy techniques to moisture is a significant challenge in grain storage and processing environments. Moisture in grains can vary greatly depending on environmental conditions, such as temperature and humidity. High moisture content can lead to the scattering of light, which disrupts the accuracy and reproducibility of spectral measurements. For example, in NIR and MIR spectroscopy, water strongly absorbs specific wavelengths, altering the spectral signal and leading to potential misinterpretation of the data. This issue is exacerbated in grain storage facilities, where humidity levels fluctuate, making real-time, on-site detection of mycotoxins difficult without controlling for moisture content. Such variability may require frequent recalibration of the spectroscopic system or complex pre-treatment techniques to mitigate moisture effects, further complicating deployment in industrial settings. Similarly, the modest penetration depth of MIR and Raman spectroscopy limits their effectiveness in detecting mycotoxins in bulk grains or thick storage materials. These techniques primarily collect data from the sample’s surface, meaning that internal contamination may go undetected unless additional sample preparation, such as grinding or homogenization, is performed. In high-throughput processing lines, this limitation presents a significant hurdle, as these methods may fail to assess contamination deep within grain samples, such as that found in thicker layers or packed materials. Furthermore, the reliance on surface analysis could lead to false negatives, where contamination is only present internally but is not detectable by the spectroscopy methods. These challenges reduce the reliability and accuracy of spectroscopy-based systems for large-scale, continuous monitoring in grain storage and processing operations.

7. Standard operating procedures and regulatory approvals for mycotoxin detection based on spectroscopic methods still need to be improved. Existing protocols emphasize calibrating with diverse datasets, moisture compensation, and consistent sample preparation to ensure reliable results. However, a lack of universally adopted guidelines across spectroscopic platforms hampers broader industrial deployment. To advance regulatory approval, it is crucial to develop standardized protocols that define key performance metrics such as sensitivity and reproducibility.

## 7. Conclusions

Vibrational spectroscopy techniques have been used to assess mycotoxin hazards in grains for grain quality monitoring. This review summarizes the research progress of several classical vibrational spectroscopy techniques in evaluating fungal contamination in grains. The results show that detecting toxins such as AFB1, AF, DON, and ZEN in grains is feasible using vibrational spectroscopy techniques. Each technique has its unique applications and characteristics. Raman and NIR spectroscopy are commonly used to analyze ground grain products. In contrast, MIR and HSI can be used to analyze individual grain kernels, although their quantitative performance still needs improvement. SERS offers extremely low detection limits, but the selection and preparation of nanomaterial substrates remain crucial. In conclusion, no single technique is perfect, and common limitations persist, requiring further attention in future research. Future research should focus on advancing data-driven intelligent modeling, developing platform-level databases, building transferable modeling systems, integrating synergistic spectral information, and optimizing device miniaturization. These efforts should aim to transform spectral detection from being merely “feasible” to truly “practical,” and extend its application from the “laboratory” to widespread “on-site” use.

## Figures and Tables

**Table 1:** The application of Vis–NIR in the detection of mycotoxins.

Samples	Toxins	Range	Processing	Modeling	Performance	Ref.
Wheat	DON	0–49.3 mg/kg	Ground Unground	Random forest (RF) Extra trees (ET) AdaBoost (AB)	Kernels: ET: R^2^ = 0.77 RF: Acc = 84.2% Flour: ET: R^2^ = 0.94 AB: Acc = 89%	[101]
Wheat	Fusarium head blight(FHB)	/	Ground Unground	Linear discriminant Analysis (LDA), RF	Kernels: Acc = 100% Flour: Acc = 100%	[97]
Maize	Aflatoxin	0–50 ng/g	Unground	LDA, PLSR	R^2^ = 0.99, Acc = 100%	[100]
Wheat	Fusarium and Aspergillus	/	Unground	LDA, PLSR	R^2^ = 0.89, Acc = 91.7%	[98]
Peanut	AFB1	10–1000 ppb	Unground	PLS-DA	20 ppb: Acc = 90% 40 ppb: Acc = 94.29%	[99]
Peanut	AFB1	1.37–268.16 ug/kg	Ground	PLS-DA, PLS	R = 0.956, Acc = 91.9%	[102]
White	AFB1	6.57–124.29 ug/kg	Unground	PLSR	>35: R^2^ = 0.69 <35: R^2^ = 0.61	[103]
Maize	Versicolorin A	/	Ground	K-nearest neighbors (KNN), SVM, XGBoost	SVM: Acc = 90% XGBoost: R^2^: 0.97	[104]

**Table 4:** The Application of Raman in the Detection of Mycotoxins.

Samples	Toxins	Range	Processing	Modeling	Performance	Ref.
Maize	AFB1	2.6214–63.0195 ug/kg	Ground	SVM	R^2^ = 0.9715	[124]
Wheat	ZEN	2–63 ug/kg	Ground	CNN	R^2^ = 0.9837	[125]
Wheat	AFB1	2.040–92.534 ug/kg	Ground	PLSR	R^2^ = 0.9927	[126]
Peanut	AFB1	2.1207–290.0161 ug/kg	Ground	PLSR	R = 0.9558	[127]
Maize	ZEN	6.90–800.20 ug/kg	Ground	PLSR	R = 0.9260	[128]
Peanut	AF	30–400 ppb	Unground	SIMCA	Acc = 80.8%	[129]

**Table 7:** Comparative analysis table of spectroscopic techniques.

Spectral Technology	Principle	Benefits	Shortcomings	Sensitivity	Sample Requirements
Vis–NIR	Color + vibration frequency multiplication/combination frequency	Fast, low cost, sensitive to color changes	Low specificity, spectral overlap	μg/kg-mg/kg	Ground, Unground
NIR	The vibration of the hydrogen-containing group is multiplied or combined	Fast, good penetration, mature, high online potential	Water effect, model dependence is strong	μg/kg	Ground, Unground
MIR	Fundamental frequency vibration	Rich molecular structure information, high specificity, molecular fingerprint interval	Shallow penetration, moisture-sensitive	μg/kg	Ground, Unground, Tablet
Raman	Inelastic scattering vibration	Water compatibility	Weak signal and prone to fluorescence interference	μg/kg	Ground, Unground
SERS	Raman scattering, surface signal enhancement	Ultra-high sensitivity, High specificity	Strong dependence on the base, challenges in reproducibility/stability, and high cost	ng/kg-pg/kg	Extraction, Base Preparation
HSI	Spectral spatial imaging	Spatial information distribution, heterogeneity analysis	Large volume of data, complex processing, and expensive instruments	μg/kg-mg/kg	Ground, Unground

## Data Availability

No new data were created or analyzed in this study.
